# Valid group comparisons can be made with the Patient Health Questionnaire (PHQ-9): A measurement invariance study across groups by demographic characteristics

**DOI:** 10.1371/journal.pone.0221717

**Published:** 2019-09-09

**Authors:** David Villarreal-Zegarra, Anthony Copez-Lonzoy, Antonio Bernabé-Ortiz, G. J. Melendez-Torres, Juan Carlos Bazo-Alvarez

**Affiliations:** 1 Instituto Peruano de Orientación Psicológica, Lima, Peru; 2 CRONICAS Center of Excellence in Chronic Diseases, Universidad Peruana Cayetano Heredia, Lima, Peru; 3 Universidad San Ignacio de Loyola, Lima, Peru; 4 Asociación Peruana de Profesionales de las Adicciones, Lima, Peru; 5 Universidad Científica del Sur, Lima, Peru; 6 Peninsula Technology Assessment Group, College of Medicine and Health, University of Exeter, Exeter, United Kingdom; 7 Instituto de Investigación, Universidad Católica Los Ángeles de Chimbote, Chimbote, Peru; 8 Methodology Research Group, Department of Primary Care and Population Health, University College London (UCL), London, United Kingdom; 9 PSYCOPERU Peruvian Research Institute of Educational and Social Psychology, Lima, Peru; University of Lleida, SPAIN

## Abstract

**Objective:**

Analyze the measurement invariance and the factor structure of the Patient Health Questionnaire-9 (PHQ-9) in the Peruvian population.

**Method:**

Secondary data analysis performed using cross-sectional data from the Health Questionnaire of the Demographic and Health Survey in Peru. Variables of interest were the PHQ-9 and demographic characteristics (sex, age group, level of education, socioeconomic status, marital status, and area of residence). Factor structure was evaluated by standard confirmatory factor analysis (CFA), and measurement invariance by multi-group CFA, using standard goodness-of-fit indices criteria for interpreting results from both CFAs. Analysis of the internal consistency (α and ω) was also pursued.

**Results:**

Data from 30,449 study participants were analyzed, 56.7% were women, average age was 40.5 years (standard deviation (SD) = 16.3), 65.9% lived in urban areas, 74.6% were married, and had 9 years of education on average (SD = 4.6). From standard CFA, a one-dimensional model presented the best fit (CFI = 0.936; RMSEA = 0.089; SRMR = 0.039). From multi-group CFA, all progressively restricted models had ΔCFI<0.01 across almost all groups by demographic characteristics. PHQ-9 reliability was optimal (α = ω = 0.87).

**Conclusions:**

The evidence presents support for the one-dimensional model and measurement invariance of the PHQ-9 measure, allowing for reliable comparisons between sex, age groups, education level, socioeconomic status, marital status, and residence area, and recommends its use within the Peruvian population.

## Background

Depression is currently one of the main causes of disability: an estimated 4.4% (322 million) people around the world suffer from depression (5.1% of women and 3.6% of men) [1]. In 2017, prevalence of depressive symptomatology in North America, Latin America, and the Caribbean was estimated at 15% (48.2 million) [1, 2]. In Peru, depression is the second greatest cause of healthy years lost (7.4 in men and 13.7 in women) [3], and is on track to be the leading cause of years lost to disability in 2030.[4] Thus, depression is a serious public health problem requiring large-scale intervention [5]. Valid, brief, and reliable instruments to assess depressive symptoms are also needed to guarantee appropriate monitoring of intervention effectiveness [6]. In particular, the use of brief instruments for the early detection of depressive symptomatology appears to be cost-effective [7]. Likewise, comparing disease burden between groups would help to establish priorities for social interventions [8].

Because depression is a latent construct and thus not strictly observable, instruments that can evaluate the different indicators of the construct in diverse populations are necessary. Depression instruments are commonly used for group comparisons (e.g., sex, age, or another condition), but comparisons are only valid if the measurement invariance for an instrument is established. For example, in the general population, an instrument of depression must identify higher scores in depressive symptoms beyond inherent characteristics of the participants, and also ensure that instrument items ‘point to’ the underlying experience of depression in similar ways between men and women [9].

This similarity can be verified by considering the four stages of measurement invariance, which require progressively stronger assumptions: configural, metric, strong and strict. Configural invariance implies that the instrument has an equal factorial structure between groups, i.e. the depression is measured with the same number of dimensions and items for each dimension in both sexes. Metric invariance implies that the instrument items contribute in a similar way in both groups, i.e. in men and women, items are equally meaningful indicators of depression. Strong invariance implies that the intercepts (or, for ordinal items, thresholds) are equivalent between both groups, or that the ‘set points’ for each item are similar between groups. Finally, strict invariance suggests that the variance of the residual measurement error for each item is equivalent in both groups [10].

Measurement invariance has been little studied in the Patient Health Questionnaire-9 (PHQ-9) [11], a self-administered version of PRIME-MD for common mental disorders that evaluates each of the nine depression criteria from the DSM-IV [12] (these criteria are maintained in the DSM-V). Many population studies on depression have used the PHQ-9 for group comparisons [13, 14]. However, because measurement invariance between groups has not been evaluated in these studies, differences between groups may reflect measurement bias rather than true differences in depression [10]. Without confirmed measurement invariance, there is no warranty that PHQ-9 measures the construct in the same way across groups, making any comparison hard to interpret [15].

Evidence on the PHQ-9’s measurement invariance according to sex is less consistent than in other group comparisons. Some studies support a strong invariance for sex comparisons [11, 16, 17], whereas other studies report weak or even no measurement invariance across sexes [18, 19], indicating that men and women could be interpreting the PHQ-9 items differently. In regard to other demographic variables, strong invariance has been evidenced across races/ethnicities [11], age, marital status, and educational level [16]. However, there are no studies on PHQ-9’s measurement invariance across urban/rural areas or socioeconomic status. Despite this scant evidence, several studies make comparisons between these groups using the PHQ-9 [13, 14]. Ultimately, the empirical evidence on the measurement invariance of the PHQ-9 across these demographic characteristics is still insufficient.

Indeed, it remains unclear if the PHQ-9 is consistently one-dimensional as originally developed [20]. Some studies have found only one underlying dimension that summarizes depressive symptoms as a whole [16, 18, 21]. However, depressive symptomatology is a multi-dimensional construct which includes cognitive, emotional, social, sexual, and other disruptions, and not all instruments have been designed for detecting and scaling each of its dimensions [22]. Other studies have identified at least two dimensions in the PHQ-9, including somatic, non-somatic or cognitive-affective dimensions [23–27]. Indeed, a recent systematic review identified that evidence on the dimensional structure of the PHQ-9 is still inconclusive [28]. Therefore, an important first step is to consider the dimensionality of the PHQ-9.

In view of the deficiencies in the evidence base relating to the PHQ-9’s psychometric properties, we sought to evaluate the measurement invariance of the PHQ-9 across groups by selected demographic characteristics, following three steps: 1) identify the most appropriate factor structure for the PHQ-9 in the Peruvian population (one-dimensional or two-dimensional model); 2) to assess PHQ-9 measurement invariance by sex, age, education level, socioeconomic status, marital status and rural-urban area; and 3) to estimate the PHQ-9 reliability.

## Materials and methods

### Study design

A secondary data analysis was conducted using data from the Peruvian Demographic and Health Survey (ENDES in Spanish), a nationally representative survey conducted annually. Since 2014, the ENDES has included a Health Questionnaire that assesses different aspects of health, such as mental health, oral health, and chronic diseases. Only cross-sectional information from 2016 ENDES Health Questionnaire was used, which is available at the website of the National Institute of Statistics and Informatics (INEI, in Spanish) [29].

ENDES design includes a two-stage random sampling technique, differentiated for rural and urban areas. In rural areas, the primary sampling units were groups of 500–2000 individuals and the secondary sampling units were the households within each of these groups. On the other hand, in urban areas, the sampling units consisted of blocks or groups of blocks with more than 2,000 individuals and an average of 140 households, and the secondary sampling units were the same as in rural settings [30].

### Participants

**Fig 1:**
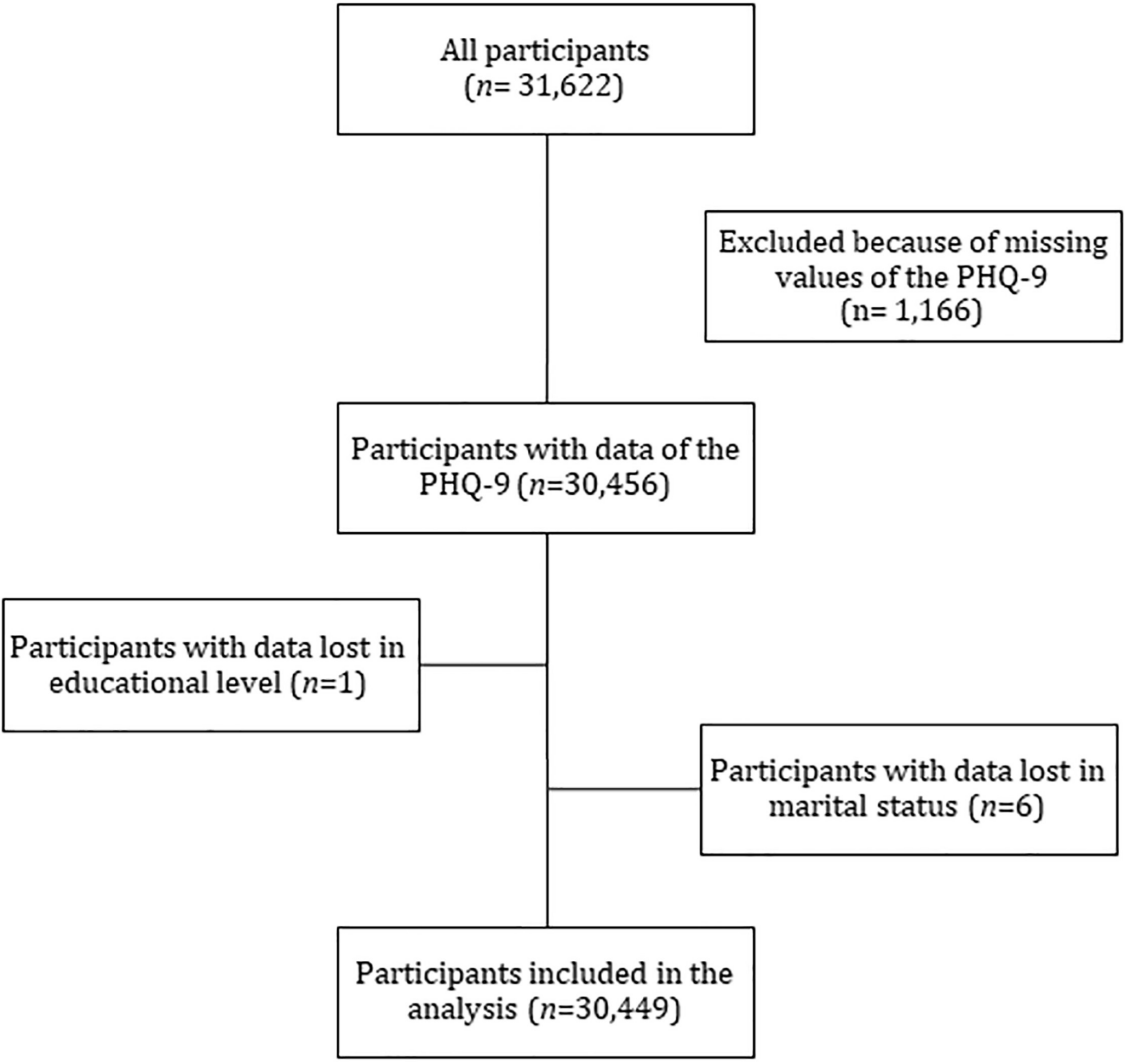
Flowchart of selected participants included in the study.

The study sample was a representative sample of the Peruvian population, made up of men and women, from urban and rural environments with multi-ethnic origins and different socioeconomic status in this middle-income country. Data from those aged ≥18 years were included, but excluding people who had missing data in the variables of interest (sex, age group, level of education, socioeconomic status, marital status, area of residence, and the nine items of PHQ). Initially there were 31,622 participants; however, after excluding participants with incomplete information, data of 30,449 participants were analyzed (see Fig 1).

### Measurements

The PHQ-9 is a Likert self-report consisting of nine items designed from the nine criteria evaluated by the DSM IV for major depression (MDD). PHQ-9 has four response options (0 = not at all; 1 = several days; 2 = more than half the days; 3 = nearly every day), with scores ranging from 0 to 27. This instrument reports the indicators of depressive symptomatology during the last two weeks.[12] In other samples, the PHQ-9 has presented adequate levels of reliability (α = 0.84) [31], and adequate levels of specificity (>0.90) but low sensitivity, between 0.39 and 0.73 [32].

Other variables were added to analyze the characteristics of the population as well as the measurement invariance of the model. These variables were: sex, education level (primary education [up to 6 years], secondary education [7–11 years] and superior [≥ 12 years]), age group [33] (young [18 to 34], early adulthood [35 to 54], intermediate adulthood [55 to 74], and older adults [≥ 75 years]), socioeconomic status (defined according to household assets and then split in tertiles [low, medium and high]), marital status (married, never married and previously married), residence area (urban and rural) and natural region (coast, mountains and jungle); the latest not used for measurement invariance analysis.

### Statistical analysis

A polychoric correlation matrix was calculated using sampling weights and used for the subsequent analysis (see S1 Table). Subsequently, a confirmatory factor analysis determined the dimensionality of the PHQ-9 in our target population. After identifying the number of dimensions, the measurement invariance was assessed to establish the PHQ-9 equivalence across groups by demographic characteristics. Finally, we performed the reliability analysis to determine the internal consistency of the PHQ-9 measures.

#### Confirmatory factor analysis

One-dimensional and two-dimensional measurement models that have been shown to be feasible for the PHQ-9 [20, 23–25] were evaluated to identify optimal fit in the target population (see M1, M2, M3, and M4 in S1 Fig). The estimator used was weighted least squares means and variance adjusted (WLSMV), which allows handling non-normality in the confirmatory factor analysis (CFA) [34].

The adjustment of the models was evaluated through two successive steps. First, the Comparative Fit Index (CFI) and the Tucker-Lewis Index (TLI), both with appropriate values ≥0.90; the Standardized Root Mean Square Residual (SRMR); and the Root Mean Square Error of Approximation (RMSEA) with a confidence interval of 90%, and with adequate values <0.08, were used to compare model fit [35, 36]. As a second and last step, the correlation between the somatic and affective-cognitive dimension was evaluated (in the case of two-dimensional models), since a very high correlation would indicate that both dimensions would be overlapping. A clear differentiation between both dimensions can be considered when the correlation is less than 0.80 [37].

#### Measurement invariance

Multiple models of the CFA measurement invariance were evaluated through groups defined by relevant variables (sex, age group, education level, socioeconomic status, marital status, and residence area). Thus, four measurement models with progressive restrictions were compared between categories of these groups (e.g. between females and males) [10, 38]. Change in the CFI (ΔCFI) was used as the main criterion for comparing models with more restrictions against models with fewer restrictions. Simulation evidence suggests that ΔCFI < .01 between successively more restricted models provides evidence for measurement invariance [10]. Models first assumed configural invariance (i.e. similar factor structure across groups) as the base model, progressing to metric invariance (i.e. similar factor loadings and factor structure across groups), strong invariance (i.e. similar thresholds, factor loadings and factor structure across groups), and strict invariance (i.e. similar residual item variances, thresholds, factor loadings and factor structure across groups). Between each model, the ΔCFI was examined to establish if the more restricted model was appropriate. We preferred ΔCFI over χ^2^ comparisons, since the first is not sensitive to big sample sizes [10, 38].

#### Reliability

Reliability analyses were performed using the classic alpha (α) and categorical omega coefficient (ω), which optimal values are> 0.80, and the item-test correlation which optimal values are >0.20 [39–41].

All analyses were done in R Studio, with the packages “lavaan” [42], “lavaan.survey” [43], “semTools” [44], and “semPlot” [45].

## Results

### Participants characteristics

The sample consisted of men (n = 13,196, 43.3%) and women (n = 17,253, 56.7%), the ages ranged from 18 to 98 years old, the mean age was 40.5 (SD = 16.3) and on average, participants had 9 years of education (SD = 4.6) (see Table 1). Likewise, the participants of our study are compared with the results of the last Peruvian census (see S2 Table).

**Table 1:** Characteristics of the participants included in the study.

		Overall		Men		Women	
		*N*	%	*n*	%	*n*	%
Score in PHQ-9	0 to 9	28,077	92.2%	12,556	95.2%	15,521	90.0%
	10 to 14	1,438	4.7%	450	3.4%	1,043	6.0%
	15 to more	934	3.1%	190	1.4%	689	4.0%
Age	18–34	13,576	44.6%	5,404	41.0%	8,172	47.4%
	35–54	10,739	35.3%	4,946	37.5%	5,793	33.6%
	55–74	4,791	15.7%	2,242	17.0%	2,549	14.8%
	75+	1,343	4.4%	604	4.5%	739	4.3%
Education level	Up to 6 years	10,314	33.9%	4,353	33.0%	5,961	34.5%
	7–11 years	12,152	39.9%	5,394	40.9%	6,758	39.2%
	12+ years	7,983	26.2%	3,449	26.1%	4,534	26.3%
Socioeconomic status	Lowest	10,280	33.8%	4,575	34.7%	5,705	33.1%
	Middle	10,213	33.5%	4,347	32.9%	5,866	34.0%
	Highest	9,956	32.7%	4,274	32.4%	5,682	32.9%
Marital status	Married	22,702	74.6%	10,410	78.9%	12,292	71.3%
	Never married	2,901	9.5%	1,293	9.8%	1,608	9.3%
	Previously married	4,846	15.9%	1,493	11.3%	3,353	19.4%
Natural region	Coastal	12,335	40.5%	5,349	40.5%	6,986	40.5%
	Highlands	10,770	35.4%	4,524	34.3%	6,246	36.2%
	Jungle	7,344	24.1%	3,323	25.2%	4,021	23.3%
Residence area	Urban	20,066	65.9%	8,524	64.6%	11,542	66.9%
	Rural	10,383	34.1%	4,672	35.4%	5,711	33.1%

### Confirmatory factor analysis

**Fig 2:**
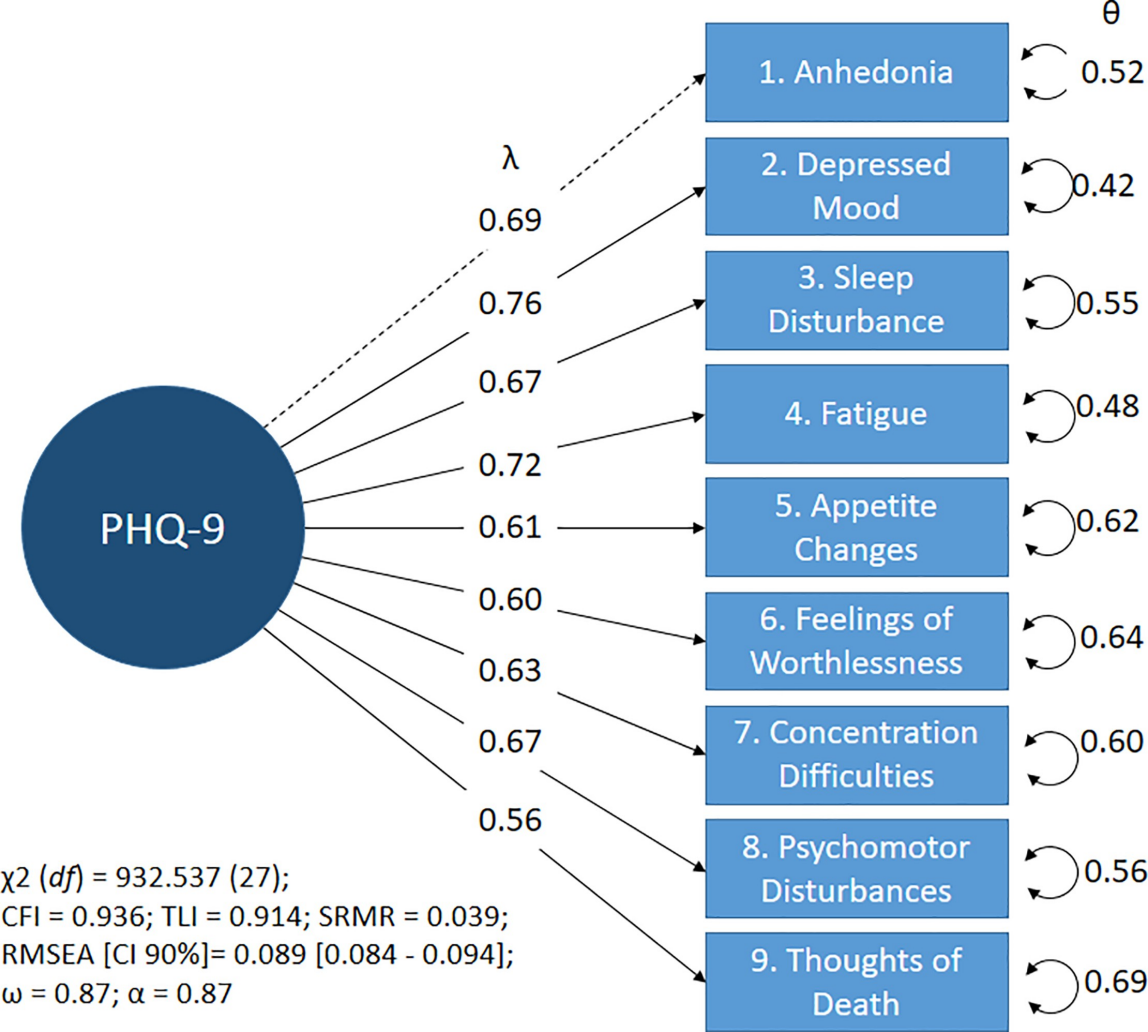
Factorial structure of Patient Health Questionnaire-9 (two weeks). Note: The circular figure indicates a latent variable; the figure of the rectangles indicates observed variables (items); λ = standardized factor loads; θ = error variance; CFI and TLI = comparative adjustment index considers optimal values ≥0.95; SRMR = Mean root of standardized error considers adequate values <0.08; RMSEA = Mean root of the approximation error considers appropriate values <0.08.

It was identified that the models of one and two dimensions present adequate indexes of goodness-of-fit (see S3 Table). However, the correlations between the dimensions in the two-factor models (somatic and cognitive-affective) ranged between 0.97 and 0.99. Therefore, the one-dimensional model was carried forward for measurement invariance testing (see Fig 2).

### Measurement invariance

The values of ΔCFI were <0.01 when all models, with progressive restrictions, were compared across age groups, sex, level of education, socioeconomic status, marital status, and residence area (see Table 2). All groups reported strict invariance.

**Table 2:** Measurement invariance in the Patient Health Questionnaire-9 (two weeks) for the different groups.

Group	Invariance	*x*^*2*^(df)	CFI	RMSEA	Δ *x*^2^(Δ df)	ΔCFI	Δ RMSEA
Sex	Configural	5284.4 (54)***	0.974	0.080 [0.078–0.082]	-	-	-
	Metric	4221.0 (62)***	0.980	0.066 [0.065–0.068]	16.8 (8)	0.005	0.013
	Strong	5408.9 (71) ***	0.974	0.070 [0.069–0.072]	38.3 (9)	0.006	0.004
	Strict	4516.0 (80) ***	0.978	0.060 [0.059–0.062]	54.4 (9)	0.004	0.010
Age	Configural	5297.4 (108)***	0.974	0.079 [0.078–0.081]	-	-	-
	Metric	3708.9 (132)***	0.982	0.060 [0.058–0.061]	35.2 (24)	0.008	0.020
	Strong	5489.2 (159)***	0.973	0.066 [0.065–0.054]	2.9 (27)	0.009	0.017
	Strict	4595.7 (186)***	0.978	0.056 [0.054–0.057]	137.3 (27)	0.005	0.011
Education	Configural	5111.9 (81)***	0.976	0.078 [0.076–0.080]	-	-	-
Level	Metric	3707.6 (97)***	0.983	0.061 [0.059–0.062]	22.8 (16)	0.007	0.017
	Strong	5165.1 (115)***	0.976	0.066 [0.064–0.067]	34.9 (18)	0.007	0.005
	Strict	4007.8 (133)***	0.982	0.054 [0.052–0.055]	32.4 (18)	0.006	0.012
Socioeconomic	Configural	5163.9 (81)***	0.976	0.079 [0.077–0.080]	-	-	-
Status	Metric	3732.4 (97)***	0.983	0.061 [0.059–0.062]	19.3 (16)	0.007	0.018
	Strong	5227.9 (115)***	0.976	0.066 [0.065–0.068]	18.4 (18)	0.007	0.005
	Strict	4244.7 (133)***	0.981	0.055 [0.054–0.057]	109.0 (18)	0.005	0.011
Marital status	Configural	5171.3 (81)***	0.975	0.079 [0.077–0.081]	-	-	-
	Metric	3678.5 (97)***	0.982	0.060 [0.059–0.062]	26.2 (16)	0.007	0.018
	Strong	5159.9 (115)***	0.975	0.066 [0.064–0.067]	43.3 (18)	0.007	0.005
	Strict	3960.5 (133)***	0.981	0.053 [0.052–0.055]	42.3 (18)	0.006	0.012
Residence area	Configural	5252.9 (54)***	0.975	0.080	-	-	-
(rural/urban)	Metric	4223.0 (62)***	0.980	0.066	20.0 (8)	0.005	0.013
	Strong	5453.0 (71)***	0.975	0.071	30.6 (9)	0.006	0.004
	Strict	4542.0 (80)***	0.979	0.061	58.5 (9)	0.004	0.0!0

Note

*** = p < .001; the results correspond to the one-dimensional model (M1).

### Reliability

The reliability of the PHQ-9 scores was high, reaching coefficients of internal consistency of α = 0.870 and ω = 0.873. On the other hand, the item-test correlation fluctuated between 0.62 and 0.77 (see Table 3).

**Table 3:** Descriptive measures and item-test correlation of the items in the Patient Health Questionnaire-9 (two weeks).

Items	SE	*M* (CI 95%)	SD	*g*1	*g*2	*r*_itc_
1. Anhedonia	0.004	0.48 (0.49–0.47)	0.77	1.74	5.59	0.729
2. Depressed mood	0.005	0.53 (0.54–0.52)	0.81	1.60	5.03	0.774
3. Sleep disturbance	0.004	0.39 (0.40–0.39)	0.78	2.11	6.77	0.708
4. Fatigue	0.004	0.35 (0.36–0.35)	0.70	2.24	7.80	0.753
5. Appetite changes	0.004	0.31 (0.32–0.30)	0.70	2.49	8.82	0.679
6. Feelings of worthlessness	0.003	0.20 (0.21–0.20)	0.57	3.32	14.50	0.668
7. Concentration difficulties	0.004	0.28 (0.29–0.27)	0.64	2.63	10.00	0.703
8. Psychomotor disturbances	0.004	0.26 (0.26–0.25)	0.62	2.80	10.94	0.718
9. Thoughts of death	0.003	0.14 (0.14–0.13)	0.48	4.16	21.80	0.636
Total	0.024	2.95 (3.00–2.90)	4.33	2.16	8.62	-

*Note*:SE = standard error; M = Mean; SD = standard deviation; g1 = skewness; g2 = kurtosis; *r*_it_ = item-test correlation; the results correspond to the one-dimensional model (M1).

## Discussion

### Main findings

The PHQ-9 showed consistently good measurement invariance, allowing comparisons between groups by age, sex, educational level, socioeconomic status, marital status, and residence area. Measurement invariance provides confidence that any difference between PHQ-9 one-dimension measures across these groups comes from a real difference in depressive symptomatology and not from group-specific properties of the instrument itself. Additionally, our evidence supported an optimal reliability of PHQ-9.

### Factorial structure

Though goodness-of-fit indices indicated that two-dimensional models fit the data better than the one-dimensional model, the correlation between these two factors ("somatic" and "cognitive-affective") was consistently very high across all models (.967 to .988). This indicates a substantial overlap between the two factors, complicating the interpretation of the results of the test [37], and pointing to the value of a more parsimonious unidimensional solution. It should be noted that the single-factor model is the most studied and used in applications of the PHQ-9 [28, 32], and indeed that the PHQ-9 was designed as a one-dimensional screening tool to evaluate the nine DSM diagnostic indicators [11]. Other evaluation instruments, such as the Beck Depression Inventory (BDI-II) and the Center for Epidemiological Studies Depression scale (CES-D), consider that depression is a multidimensional construct and evaluate additional items of sexual problems, indecision, self-criticism, feelings of anxiety, among others. These instruments’ additional dimensions does not imply that the PHQ-9 collects partial information on the construct, since these additional indicators are not part of the main diagnostic criteria for major depression disorder. Several studies conducted in the general population and primary care support the one-dimensional model of the PHQ-9. For instance, a study in primary care centers in Spain (n = 836), in primary care patients with different ethnic origins and risk of depression from the Netherlands (n = 1,772), and another in the general population of Hong Kong (n = 6,028), coincide with our findings that the one-dimensional model of the PHQ-9 is the most parsimonious and stable [16, 18, 21].

However, two studies in American samples have found two dimensions: one study using a representative sample (n = 26,202) and one study in a sample of soldiers (n = 2,615) [11, 26]. Yet the relationship between the two factors was very high (0.87 in both cases). Both studies coincide with our results, suggesting an overlap between both dimensions. On the other hand, a German study in patients with major depression (n = 626) and another in cancer patients with palliative care from the UK (n = 300) [25, 27], identified the somatic and affective-cognitive dimensions as related but distinct, with a correlation between the latent dimensions of 0.58 and 0.30, respectively. A possible explanation for the heterogeneity of the results on the internal structure of the PHQ-9 is the population evaluated. Investigations that report a clear differentiation between the somatic and cognitive-affective dimensions predominantly draw on clinical populations [25, 27], whereas those that report an overlap between both dimensions are performed in the general population [11, 26]. Living several years with depression or with a chronic disease that significantly affects physical health could cause people to differentiate physical or somatic indicators from those affective-cognitive. For example, it is possible that cancer patients might have a high score on the items on sleep disturbance, fatigue, and appetite changes (often associated with the somatic dimension), because these are side effects of treatment, but score low on cognitive-affective items. This would diminish the correlation between the two dimensions and give rise to the appearance of differentiation. In terms of behavior, the dimensionality of the detection of depressive symptoms would be mediated by contingencies associated with physical comorbidity [16]. Finally, it is possible that cultural factors play a role in whether the depressive symptomatology is perceived as a single construct, or as two related elements (somatic and cognitive-affective) [46]. However, it is not possible to identify what might be the psychological mechanisms that would generate an overlap between the two dimensions in these culturally different studies.

Likewise, it is necessary to point out the practical disadvantages generated by having a model of two subdimensions instead of a one-dimensional model. In addition, the original qualification method is based on a one-dimensional model that sum up the direct scores of all items.[12] It should be noted that the original cutoff points for determining levels of morbidity (≤5, ≤10, ≤15 and ≤20) have proven to be more appropriate compared to alternative classification methods [32].

### Measurement invariance

Our results support that the PHQ-9 presents convincing measurement invariance in the groups of age, sex, educational level, socioeconomic status, residence area, and marital status, allowing meaningful group comparisons. Other studies, in university students from the United States (n = 857) and primary care in Spain (n = 836), support our results as they report strong measurement invariance according to sex [16, 17]. On the other hand, a study in primary care patients at high risk of depression in the Netherlands identified that the measurement invariance at the level of factor loadings was violated [18] because women presented higher loadings for "sleep disturbance" and men for "loss of interest". These results suggest that the measurement invariance between men and women is not met in a population at high risk of depression or depression. That is to say, as the depressive symptomatology increases, the differences between both sexes accrue, meaning that women and men tend to score higher in a different group of items (i.e. sleep disturbance and loss of interest). Different studies also report that the prevalence of sleep and appetite problems is greater in women than in men [47, 48]. Our results support that it is possible to make comparisons between men and women in the Peruvian population. Similarly, accumulated international evidence supports the possibility of making comparisons between men and women using the one-dimensional model in the general population [49].

Our results support the presence of a strong invariance according to the age, educational level, and marital status, allowing comparisons between groups in the Peruvian population. A Spanish study conducted in a small group of primary care patients also found strong invariance between age groups, marital status, and educational level [16]. At the level of measurement invariance between age groups, there is evidence that depression among young people and older adults is qualitatively different, in addition to the fact that older adults have a higher prevalence of depressive symptoms [48]. However, this does not seem to affect the factor structure or how they understand the construct. With respect to measurement invariance by educational level and marital status, it is not possible to identify a plausible psychological or biological mechanism that could justify a possible violation of invariance. However, these analyzes are necessary because of their impact on practice since several studies make comparisons between these groups [13, 14]. Our results, like those developed in the Spanish study, support making comparisons validity between age, educational level, and marital status.

The present study supports the possibility of making comparisons between socioeconomic status and area of residence (urban and rural); however, our results could not be compared with other studies since no other research was found that evaluated the measurement invariance according to these groups using the PHQ-9. Despite the limited evidence on measurement invariance in these groups, different studies have already made comparisons between the direct scores of the PHQ-9 in people from urban and rural areas, as well as between people of different socioeconomic status [14]. Theories of social disadvantage and structural determinants of health could explain a possible difference between direct scores, due to limited access to opportunities and limited access to specialized health services [50].

### Reliability

Our results present optimal reliability coefficients of the scores derived from the measure of the PHQ-9 in the Peruvian population. This is consistent with other findings in the literature. In particular, two studies with a similar sample size carried out in the general population of China [21] and Germany [20], identified very similar values of internal consistency (classical alpha) of 0.82 and 0.86, respectively. Therefore, despite being studies in culturally different populations, the measurements of depression symptoms using the PHQ-9 present a similar internal consistency. Some differences in the characteristics of the participants of the ENDES and the Peruvian census of 2017 are identified, especially in the level of education, marital status, natural region, and area of residence. This may be because ENDES was originally designed to be representative of women of fertile age. This would be generating that the characteristics of the participants differ between ENDES and the Peruvian census. It should be noted that this should not affect the conclusions of the study.

### Relevance in public health

The PHQ-9 is one of the instruments most used by researchers and mental health professionals around the world to evaluate depressive symptoms [32]. Within its applications in public health, its use is recommended to evaluate depressive symptomatology in clinical trials and research in general [24, 32], since it is an instrument with solid evidence of validity and reliability. Likewise, different countries promote its use in primary care [51, 52], owing to its brevity, easy scoring (add the nine items), and applicability across heterogeneous sociodemographic characteristics. Our results support the use of the Spanish version of the PHQ-9 in the Peruvian population. These evidences suggest that it is possible to use the PHQ-9 in other Spanish-speaking countries in Latin America.

### Strengths and limitations

Among the strengths of the study are the large sample size and the representativeness of the study sample. This is the only study reported in Latin America that evaluates the measurement invariance of PHQ-9. However, the study is not free of limitations. Our results and conclusions are focused on the Peruvian population, so, results can be extrapolated with caution towards potentially similar populations. Additionally, neither the inter-rater effect (different level of experience of the interviewers) nor the inter-family effect (participants from the same family group) was controlled. However, this should not change our results, since all the evaluators received intensive training for several weeks before conducting the evaluations, so it is expected that such training will homogenize the evaluation process and gather information [30]. On the other hand, it is expected that although there are cases where two or more participants belong to the same family group, this number should be minimal compared to the total evaluated. Despite these limitations, our results are still valid and reliable.

## Conclusions

The evidence presents support for the one-dimensional model and measurement invariance of the PHQ-9 measure, allowing for reliable comparisons between sex, age groups, education level, socioeconomic status, marital status, and residence area, and recommends its use within the Peruvian population.

## Supporting information

S1 Fig Structure of the eight models to be evaluated. (DOCX)

S1 Table Polychoric correlation matrix considering the weighting for complex samples for the Patient Health Questionnaire-9 (two weeks). (DOCX)

S2 Table Comparison between the results of our study and the results of the Peruvian census of 2017, only people with age of 18 or more were considered. (DOCX)

S3 Table Confirmatory factor analysis and reliability in the Patient Health Questionnaire-9 (two weeks). (DOCX)

## References

[1] World Health Organization. Depression and Other Common Mental Disorders Global Health Estimates. Geneve: World Health Organization; 2017.

[2] Marcus M, Yasamy MT, van Ommeren M, Chisholm D, Saxena S. Depression: A global public health concern. WHO Department of Mental Health and Substance Abuse. 2012;1:6–8.

[3] Ministerio de Salud del Perú. Estudio de Carga de Enfermedad en el Perú - 2004. Perú: Ministerio de Salud del Perú; 2006.

[4] World Health Organization. The global burden of disease: 2004 update. Geneva: WHO; 2008.

[5] Boyd RC, Butler L, Benton TD. Understanding Adolescents’ Experiences with Depression and Behavioral Health Treatment. The Journal of Behavioral Health Services & Research. 2018;45(1):105–11. 10.1007/s11414-017-9558-7 28488156

[6] Williams JW, Pignone M, Ramirez G, Stellato CP. Identifying depression in primary care: a literature synthesis of case-finding instruments. General hospital psychiatry. 2002;24(4):225–37. 12100833 10.1016/s0163-8343(02)00195-0

[7] Jiao B, Rosen Z, Bellanger M, Belkin G, Muennig P. The cost-effectiveness of PHQ screening and collaborative care for depression in New York City. PloS one. 2017;12(8):e0184210 10.1371/journal.pone.0184210 28859154 PMC5578679

[8] Gonzalez-Pier E, Gutierrez-Delgado C, Stevens G, Barraza-Llorens M, Porras-Condey R, Carvalho N, et al Priority setting for health interventions in Mexico's System of Social Protection in Health. Lancet (London, England). 2006;368(9547):1608–18. Epub 2006/11/07. 10.1016/s0140-6736(06)69567-6 . 17084761

[9] Byrne BM. Structural equation modeling with EQS: Basic concepts, applications, and programming, second edition: Taylor and Francis; 2013. 1–440 p.

[10] Putnick DL, Bornstein MH. Measurement invariance conventions and reporting: The state of the art and future directions for psychological research. Developmental Review. 2016;41:71–90. 10.1016/j.dr.2016.06.004 27942093 PMC5145197

[11] Patel JS. Measurement Invariance of the Patient Health Questionnaire-9 (Phq-9) Depression Screener in U.S. Adults Across Sex, Race/Ethnicity, and Education Level: Nhanes 2005–2014. EEUU: Purdue University; 2017. 10.1002/da.22940 PMC6736700 31356710

[12] Spitzer RL, Kroenke K, Williams JB. Validation and utility of a self-report version of PRIME-MD: the PHQ primary care study. JAMA. 1999;282(18):1737–44. 10.1001/jama.282.18.1737 10568646

[13] Yu S, Yang H, Guo X, Zheng L, Sun Y. Metabolic syndrome and depressive symptoms among rural Northeast general population in China. BMC public health. 2017;17(1):43 Epub 2017/01/08. 10.1186/s12889-016-3913-0 28061774 PMC5219740

[14] Choi GS, Shin YS, Kim JH, Choi SY, Lee SK, Nam YH, et al Prevalence and risk factors for depression in Korean adult patients with asthma: is there a difference between elderly and non-elderly patients? Journal of Korean medical science. 2014;29(12):1626–31. Epub 2014/12/04. 10.3346/jkms.2014.29.12.1626 25469061 PMC4248582

[15] Gregorich SE. Do Self-Report Instruments Allow Meaningful Comparisons Across Diverse Population Groups? Testing Measurement Invariance Using the Confirmatory Factor Analysis Framework. Medical care. 2006;44(11 Suppl 3):S78–S94. 10.1097/01.mlr.0000245454.12228.8f PubMed PMID: PMC1808350. 17060839 PMC1808350

[16] Gonzalez-Blanch C, Medrano LA, Munoz-Navarro R, Ruiz-Rodriguez P, Moriana JA, Limonero JT, et al Factor structure and measurement invariance across various demographic groups and over time for the PHQ-9 in primary care patients in Spain. PloS one. 2018;13(2):e0193356 Epub 2018/02/24. 10.1371/journal.pone.0193356 29474410 PMC5825085

[17] Keum BT, Miller MJ, Inkelas KK. Testing the factor structure and measurement invariance of the PHQ-9 across racially diverse U.S. college students. Psychol Assess. 2018 Epub 2018/03/23. 10.1037/pas0000550 . 29565614

[18] Baas KD, Cramer AO, Koeter MW, van de Lisdonk EH, van Weert HC, Schene AH. Measurement invariance with respect to ethnicity of the Patient Health Questionnaire-9 (PHQ-9). J Affect Disord. 2011;129(1–3):229–35. Epub 2010/10/05. 10.1016/j.jad.2010.08.026 . 20888647

[19] Galenkamp H, Stronks K, Snijder MB, Derks EM. Measurement invariance testing of the PHQ-9 in a multi-ethnic population in Europe: the HELIUS study. BMC psychiatry. 2017;17(1):349 Epub 2017/10/27. 10.1186/s12888-017-1506-9 29065874 PMC5655879

[20] Kocalevent RD, Hinz A, Brahler E. Standardization of the depression screener patient health questionnaire (PHQ-9) in the general population. Gen Hosp Psychiatry. 2013;35(5):551–5. Epub 2013/05/15. 10.1016/j.genhosppsych.2013.04.006 . 23664569

[21] Yu X, Tam WW, Wong PT, Lam TH, Stewart SM. The Patient Health Questionnaire-9 for measuring depressive symptoms among the general population in Hong Kong. Comprehensive psychiatry. 2012;53(1):95–102. Epub 2011/01/05. 10.1016/j.comppsych.2010.11.002 . 21193179

[22] Vares EA, Salum GA, Spanemberg L, Caldieraro MA, Fleck MP. Depression Dimensions: Integrating Clinical Signs and Symptoms from the Perspectives of Clinicians and Patients. PloS one. 2015;10(8):e0136037 10.1371/journal.pone.0136037 26313556 PMC4552383

[23] Granillo MT. Structure and function of the Patient Health Questionnaire-9 among Latina and non-Latina White female college students. Journal of the society for social work and Research. 2012;3(2):80–93.

[24] Guo B, Kaylor-Hughes C, Garland A, Nixon N, Sweeney T, Simpson S, et al Factor structure and longitudinal measurement invariance of PHQ-9 for specialist mental health care patients with persistent major depressive disorder: Exploratory Structural Equation Modelling. J Affect Disord. 2017;219:1–8. Epub 2017/05/16. 10.1016/j.jad.2017.05.020 . 28501679 PMC6602881

[25] Chilcot J, Rayner L, Lee W, Price A, Goodwin L, Monroe B, et al The factor structure of the PHQ-9 in palliative care. J Psychosom Res. 2013;75(1):60–4. Epub 2013/06/12. 10.1016/j.jpsychores.2012.12.012 . 23751240

[26] Elhai JD, Contractor AA, Tamburrino M, Fine TH, Prescott MR, Shirley E, et al The factor structure of major depression symptoms: a test of four competing models using the Patient Health Questionnaire-9. Psychiatry Res. 2012;199(3):169–73. Epub 2012/06/16. 10.1016/j.psychres.2012.05.018 . 22698261

[27] Petersen JJ, Paulitsch MA, Hartig J, Mergenthal K, Gerlach FM, Gensichen J. Factor structure and measurement invariance of the Patient Health Questionnaire-9 for female and male primary care patients with major depression in Germany. J Affect Disord. 2015;170:138–42. Epub 2014/09/23. 10.1016/j.jad.2014.08.053 . 25240840

[28] van Dijk SEM, Adriaanse MC, van der Zwaan L, Bosmans JE, van Marwijk HWJ, van Tulder MW, et al Measurement properties of depression questionnaires in patients with diabetes: a systematic review. Qual Life Res. 2018;27(6):1415–30. 10.1007/s11136-018-1782-y 29396653 PMC5951879

[29] Instituto Nacional de Estadistica e Informatica. Microdatos: Base de datos de la Encuesta Demografica y de Salud Familiar—ENDES Perú: Instituto Nacional de Estadistica e Informatica; 2017 Available from: http://iinei.inei.gob.pe/microdatos/.

[30] Instituto Nacional de Estadística e Informática. Perú: encuesta demográfica y de salud familiar ENDES 2014. Perú: Instituto Nacional de Estadística e Informática; 2015.

[31] Baader MT, Molina FJL, Venezian BS, Rojas CC, Farías SR, Fierro-Freixenet C, et al Validación y utilidad de la encuesta PHQ-9 (Patient Health Questionnaire) en el diagnóstico de depresión en pacientes usuarios de atención primaria en Chile. Rev Chil Neuropsiquiatr. 2012;50:10–22.

[32] Manea L, Gilbody S, McMillan D. A diagnostic meta-analysis of the Patient Health Questionnaire-9 (PHQ-9) algorithm scoring method as a screen for depression. General hospital psychiatry. 2015;37(1):67–75. Epub 2014/12/03. 10.1016/j.genhosppsych.2014.09.009 . 25439733

[33] Papalia DE, Villamizar S, Alberto G. Desarrollo humano: con aportaciones para Iberoamérica 1997.

[34] Brown TA. Confirmatory factor analysis for applied research. Second ed New York: The Guilford Press; 2015.

[35] Hair JF, Anderson RE, Tatham RL, Black WC. Análisis multivariante: Prentice Hall Madrid; 1999.

[36] Hu L-t, Bentler PM. Fit indices in covariance structure modeling: Sensitivity to underparameterized model misspecification. Psychological methods. 1998;3(4):424.

[37] Brown TA. Confirmatory factor analysis for applied research: Guilford Publications; 2014.

[38] Widaman KF, Reise SP. Exploring the measurement invariance of psychological instruments: Applications in the substance use domain. The science of prevention: Methodological advances from alcohol and substance abuse research. 1997:281–324.

[39] McDonald RP. Test theory: A unified treatment. New York: Taylor & Francis Group; 1999.

[40] Ten Berge JM, Sočan G. The greatest lower bound to the reliability of a test and the hypothesis of unidimensionality. Psychometrika. 2004;69(4):613–25.

[41] Kelley K, Pornprasertmanit S. Confidence intervals for population reliability coefficients: Evaluation of methods, recommendations, and software for composite measures. Psychol Methods. 2016;21(1):69–92. 10.1037/a0040086 . 26962759

[42] Rosseel Y. lavaan: An R Package for Structural Equation Modeling. Journal of statistical software. 2012;48(2):36 Epub 2012-05-24. 10.18637/jss.v048.i02

[43] Oberski D. lavaan.survey: An R Package for Complex Survey Analysis of Structural Equation Models. Journal of statistical software. 2014;57(1):27 Epub 2014-03-13. 10.18637/jss.v057.i01

[44] Jorgensen TD, Pornprasertmanit S, Schoemann AM, Rosseel Y. semTools: Useful tools for structural equation modeling: R package version 0.5–1; 2018 Available from: https://CRAN.R-project.org/package = semTools.

[45] Epskamp S. semPlot: Unified visualizations of structural equation models. Structural Equation Modeling. 2015;22(3):474–83. 10.1080/10705511.2014.937847

[46] Escovar EL, Craske M, Roy-Byrne P, Stein MB, Sullivan G, Sherbourne CD, et al Cultural influences on mental health symptoms in a primary care sample of Latinx patients. Journal of anxiety disorders. 2018;55:39–47. Epub 2018/03/27. 10.1016/j.janxdis.2018.03.005 29576380 PMC5918638

[47] Frazier L, Yu E, Sanner J, Liu F, Udtha M, Cron S, et al Gender Differences in Self-Reported Symptoms of Depression among Patients with Acute Coronary Syndrome. Nurs Res Pract. 2012;2012:109251 10.1155/2012/109251 22567222 PMC3337485

[48] Altemus M, Sarvaiya N, Neill Epperson C. Sex differences in anxiety and depression clinical perspectives. Frontiers in neuroendocrinology. 2014;35(3):320–30. Epub 2014/06/03. 10.1016/j.yfrne.2014.05.004 24887405 PMC4890708

[49] Doi S, Ito M, Takebayashi Y, Muramatsu K, Horikoshi M. Factorial validity and invariance of the Patient Health Questionnaire (PHQ)-9 among clinical and non-clinical populations. PloS one. 2018;13(7):e0199235 Epub 2018/07/20. 10.1371/journal.pone.0199235 30024876 PMC6053131

[50] Cockerham WC, Hamby BW, Oates GR. The Social Determinants of Chronic Disease. Am J Prev Med. 2017;52(1s1):S5–s12. Epub 2016/12/19. 10.1016/j.amepre.2016.09.010 27989293 PMC5328595

[51] Instituto de Evaluación de Tecnologías en Salud e Investigación. Guía de Práctica Clínica de Rehabilitación Cardiaca. Perú: EsSalud; 2018.

[52] Ministerio de Salud. Guía clínica depresión en personas de 15 años y más. Chile: Ministerio de Salud; 2013.

