# Supplementary figures and images for: Valid group comparisons can be made with the Patient Health Questionnaire (PHQ-9): A measurement invariance study across groups by demographic characteristics
Source: PLoS One. 2019 Sep 9;14(9):e0221717. doi: 10.1371/journal.pone.0221717 (PMC6733536; doi:10.1371/journal.pone.0221717)

S1 Fig.

*Structure of the eight models to be evaluated.*

*
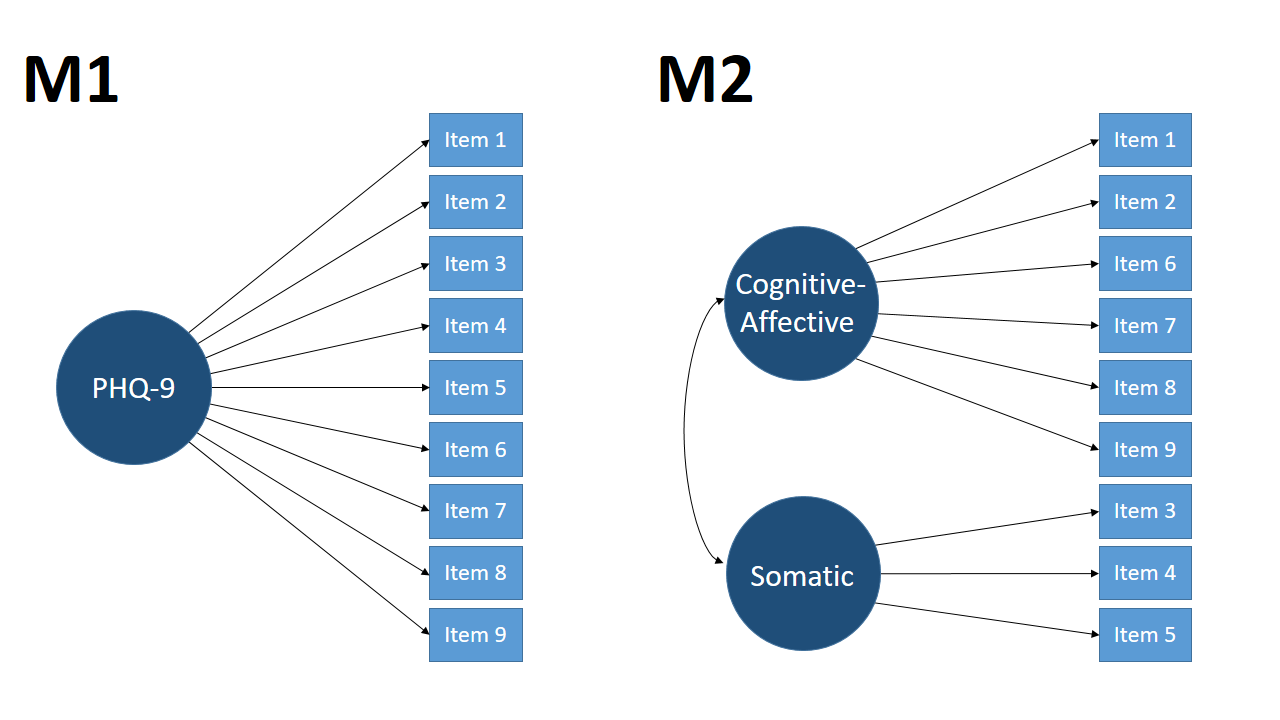

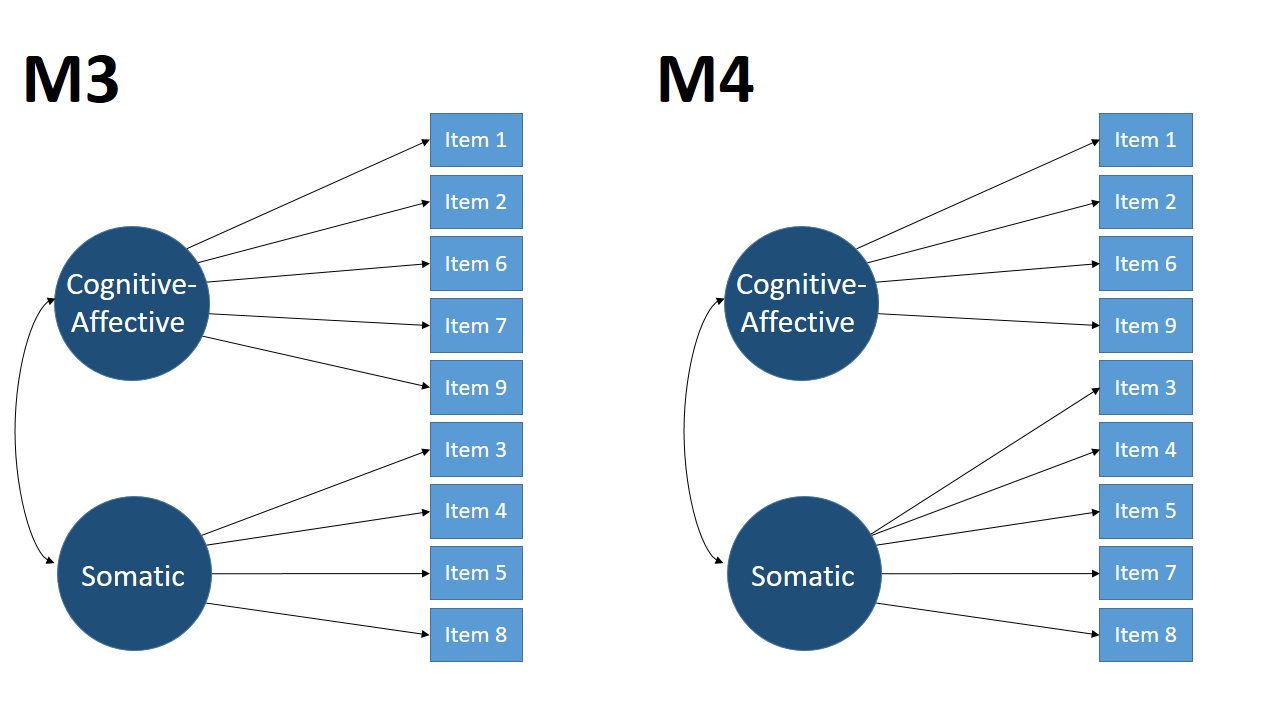
*

Supplement: S1 Fig — (DOCX) [file pone.0221717.s001.docx]
