# Supplementary material for: Valid group comparisons can be made with the Patient Health Questionnaire (PHQ-9): A measurement invariance study across groups by demographic characteristics
Source: PLoS One. 2019 Sep 9;14(9):e0221717. doi: 10.1371/journal.pone.0221717 (PMC6733536; doi:10.1371/journal.pone.0221717)
Supplement: S1 Table — (DOCX) [file pone.0221717.s002.docx]

#### *S1 Table.*

#### *Polychoric correlation matrix considering the weighting for complex samples for the Patient Health Questionnaire-9 (two weeks)*

|  | Item 1 | Item 2 | Item 3 | Item 4 | Item 5 | Item 6 | Item 7 | Item 8 | Item 9 |
| --- | --- | --- | --- | --- | --- | --- | --- | --- | --- |
| Item 1 | 1 |  |  |  |  |  |  |  |  |
| Item 2 | 0.599 | 1 |  |  |  |  |  |  |  |
| Item 3 | 0.473 | 0.515 | 1 |  |  |  |  |  |  |
| Item 4 | 0.532 | 0.543 | 0.500 | 1 |  |  |  |  |  |
| Item 5 | 0.414 | 0.439 | 0.434 | 0.447 | 1 |  |  |  |  |
| Item 6 | 0.353 | 0.449 | 0.369 | 0.418 | 0.334 | 1 |  |  |  |
| Item 7 | 0.396 | 0.458 | 0.414 | 0.443 | 0.426 | 0.375 | 1 |  |  |
| Item 8 | 0.435 | 0.468 | 0.424 | 0.470 | 0.442 | 0.411 | 0.506 | 1 |  |
| Item 9 | 0.319 | 0.426 | 0.345 | 0.375 | 0.309 | 0.549 | 0.333 | 0.391 | 1 |
