# Supplementary material for: Valid group comparisons can be made with the Patient Health Questionnaire (PHQ-9): A measurement invariance study across groups by demographic characteristics
Source: PLoS One. 2019 Sep 9;14(9):e0221717. doi: 10.1371/journal.pone.0221717 (PMC6733536; doi:10.1371/journal.pone.0221717)
Supplement: S2 Table — (DOCX) [file pone.0221717.s003.docx]

S2 Table.

*Comparison between the results of our study and the results of the Peruvian census of 2017, only people with age of 18 or more were considered.*

|  |  | Our study | Census 2017 |
| --- | --- | --- | --- |
|  |  | % | % |
| Sex | Men | 43.3% | 48.5% |
|  | Women | 56.7% | 51.5% |
| Age | 18-34 | 44.6% | 40.3% |
|  | 35-54 | 35.3% | 36.1% |
|  | 55-74 | 15.7% | 18.5% |
|  | 75+ | 4.4% | 5.1% |
| Education level | Up to 6 years | 33.9% | 25.9% |
|  | 7-11 years | 39.9% | 38.0% |
|  | 12+ years | 26.2% | 36.1% |
| Marital status | Married | 74.6% | 60.0% |
|  | Never married | 9.5% | 29.5% |
|  | Previously married | 15.9% | 10.5% |
| Natural region | Coastal | 40.5% | 58.0% |
|  | Highlands | 35.4% | 28.1% |
|  | Jungle | 24.1% | 13.9% |
| Residence area | Urban | 65.9% | 81.1% |
|  | Rural | 34.1% | 18.9% |

*Note:* The SES was not considered as it was created based on tertiles. The data of the Peruvian census of 2017 were obtained from <http://censos2017.inei.gob.pe/redatam/>.
