# Supplementary material for: Valid group comparisons can be made with the Patient Health Questionnaire (PHQ-9): A measurement invariance study across groups by demographic characteristics
Source: PLoS One. 2019 Sep 9;14(9):e0221717. doi: 10.1371/journal.pone.0221717 (PMC6733536; doi:10.1371/journal.pone.0221717)
Supplement: S3 Table — (DOCX) [file pone.0221717.s004.docx]

S3 Table.

*Confirmatory factor analysis and reliability in the Patient Health Questionnaire-9 (two weeks)*

| PHQ-9 Model | χ^2^ (*df*) | CFI | RMSEA (90%CI) | TLI | SRMR | ω_PHQ-9_ (α) | ω_somatic_ (α) | ω_affective_ (α) |
| --- | --- | --- | --- | --- | --- | --- | --- | --- |
| M1 | 932.537 (27)*** | 0.936 | 0.089 (0.084 - 0.094) | 0.914 | 0.039 | 0.87 (0.87) | - | - |
| M2 | 906.883 (26)*** | 0.937 | 0.090 (0.085 - 0.095) | 0.913 | 0.039 | - | 0.72 (0.71) | 0.83 (0.81) |
| M3 | 917.644 (26)*** | 0.937 | 0.090 (0.085 - 0.095) | 0.912 | 0.039 | - | 0.77 (0.77) | 0.80 (0.78) |
| M4 | 837.684 (26)*** | 0.942 | 0.086 (0.081 - 0.091) | 0.920 | 0.037 | - | 0.80 (0.80) | 0.78 (0.75) |

*Note:* ****p* <.001; *df* = Degrees of freedom; CFI and TLI = considering optimum values ≥0.95; SRMR and RMSEA = considering adequate values <0.08; M1= one-dimensional model;

M2= Two dimensional model with a somatic dimension (item 3, 4 and 5) a and cognitive-affective dimesion; M3= Two dimensional model with a somatic dimension (item 3, 4, 5 and 8) and cognitive-affective dimesion; M47= two-dimensional model with a somatic dimension (item 3, 4, 5, 7 and 8) and cognitive-affective dimesion.
